# Recommendations for the conduct of clinical trials for drugs to treat or prevent sarcopenia

**DOI:** 10.1007/s40520-015-0517-y

**Published:** 2015-12-30

**Authors:** Jean-Yves Reginster, Cyrus Cooper, René Rizzoli, John A. Kanis, Geoff Appelboom, Ivan Bautmans, Heike A. Bischoff-Ferrari, Maarten Boers, Maria Luisa Brandi, Olivier Bruyère, Antonio Cherubini, Bruno Flamion, Roger A. Fielding, Andrea Ildiko Gasparik, Luc Van Loon, Eugene McCloskey, Bruce H. Mitlak, Alberto Pilotto, Suzanne Reiter-Niesert, Yves Rolland, Yannis Tsouderos, Marjolein Visser, Alfonso J. Cruz-Jentoft

**Affiliations:** Department of Public Health, Epidemiology and Health Economics, University of Liege, CHU Sart Tilman B23, 4000 Liège, Belgium; MRC Lifecourse Epidemiology Unit, University of Southampton, Southampton, UK; NIHR Musculoskeletal Biomedical Research Unit, University of Oxford, Oxford, UK; Service of Bone Diseases, Geneva University Hospitals and Faculty of Medicine, Geneva, Switzerland; Centre for Metabolic Bone Diseases, and Centre for Integrated Research in Musculoskeletal Ageing, University of Sheffield, Sheffield, UK; Columbia University Medical Center, The Neurological Institute, New York, USA; Gerontology and Frailty in Ageing Research Department, Vrije Universiteit Brussel (VUB), Brussels, Belgium; Department of Geriatrics, University Hospital of Zurich, Zurich, Switzerland; Department of Epidemiology and Biostatistics; and Amsterdam Rheumatology and Immunology Center, VU University Medical Center, Amsterdam, Netherlands; Department of Internal Medicine, University of Florence, Florence, Italy; Geriatrics and Geriatric Emergency Care, IRCCS-INRCA, Ancona, Italy; Research Unit of Molecular Physiology (URPHYM), NARILIS, University of Namur, Namur, Belgium; Nutrition, Exercise Physiology and Sarcopenia Laboratory, Jean Mayer USDA Human Nutrition Research Center on Aging at Tufts University, Boston, USA; Department of Public Health and Health Management, University of Medicine and Pharmacy of Tirgu Mures, Tirgu Mures, Romania; NUTRIM School for Nutrition, Toxicology and Metabolism, Maastricht University, Maastricht, Netherlands; Lilly Research Laboratories, Eli Lilly and Company, Indianapolis, USA; Department of OrthoGeriatrics, Rehabilitation and Stabilization, Frailty Area, E.O. Galliera Hospital, NR-HS, Genoa, Italy; Bonn, Germany; Gérontopôle of Toulouse, University of Toulouse III, CHU Purpan, Toulouse, France; Institut de Recherches Internationales Servier, Suresnes, France; Department of Health Sciences, VU University, Amsterdam, Netherlands; Department of Nutrition and Dietetics, Internal Medicine, VU University Medical Center, Amsterdam, Netherlands; Servicio de Geriatría, Hospital Universitario Ramón y Cajal, Madrid, Spain

**Keywords:** Clinical trials, Sarcopenia, Public health, Preventative health care, Frailty

## Abstract

**Purpose:**

Sarcopenia is an age-related muscle condition which is frequently a precursor of frailty, mobility disability and premature death. It has a high prevalence in older populations and presents a considerable social and economic burden. Potential treatments are under development but, as yet, no guidelines support regulatory studies for new drugs to manage sarcopenia. The objective of this position paper is therefore to suggest a set of potential endpoints and target population definitions to stimulate debate and progress within the medico-scientific and regulatory communities.

**Methods:**

A multidisciplinary expert working group was hosted by the European Society for Clinical and Economic Aspects of Osteoporosis and Osteoarthritis, which reviewed and discussed the recent literature from a perspective of clinical experience and guideline development. Relevant parallels were drawn from the development of definition of osteoporosis as a disease and clinical assessment of pharmaceutical treatments for that indication.

**Results:**

A case-finding decision tree is briefly reviewed with a discussion of recent prevalence estimations of different relevant threshold values. The selection criteria for patients in regulatory studies are discussed according to the aims of the investigation (sarcopenia prevention or treatment) and the stage of project development. The possible endpoints of such studies are reviewed and a plea is made for the establishment of a core outcome set to be used in all clinical trials of sarcopenia.

**Conclusions:**

The current lack of guidelines for the assessment of new therapeutic treatments for sarcopenia could potentially hinder the delivery of effective medicines to patients at risk.

**Electronic supplementary material:**

The online version of this article (doi:10.1007/s40520-015-0517-y) contains supplementary material, which is available to authorized users.

## Introduction

Sarcopenia is a syndrome characterised by progressive and generalized loss of skeletal muscle mass and strength; it is a major pathway leading to physical frailty [1]. Since, the loss of muscle mass and strength is inherent in normal ageing, the switch towards a pathological condition must be established empirically, by the combination of diagnostic thresholds and associated risk of mobility-related outcomes, poor quality of life and death [2–4]. Until recently, there have been several different definitions of sarcopenia; a situation which may have hindered the development of regulatory and treatment guidelines. While numerous research centres are trying to develop either pharmaceutical agents, specific oral nutritional supplements or specific exercise regimes to try to counteract muscle decline [5], this lack of consensus on diagnosis, and consequently guidelines, is likely to cause inefficiencies in time and resources. To some extent, this situation resembles that of the early 1990’s when companies were trying to develop products for osteoporosis.

Following on from previous publications on the subject of sarcopenia [6–8], this position paper describes the conclusions made during an expert working group meeting of the European Society for Clinical and Economic Aspects of Osteoporosis and Osteoarthritis (ESCEO).

## The theoretical definition of sarcopenia

In 2010, three separate expert panels [2–4] reached consensus on similar statements defining sarcopenia as a condition characterised by declining muscle mass and function. In the words of the European Working Group on Sarcopenia in Older People (EWGSOP), sarcopenia is a condition ‘characterized by progressive and generalized loss of skeletal muscle mass and strength, with a risk of adverse outcomes such as physical disability, poor quality of life and death’ [2]. A key feature of this definition is the incorporation of low muscle strength (frequently referred to as dynapenia), which is generally more strongly associated with poor function and disability than low muscle mass [9]. Primary sarcopenia is then the loss of muscle mass and function that deviates negatively from normal ageing in a progressive and chronic fashion and without other obvious causal factors. Secondary sarcopenia may be used to describe the loss of muscle mass and function when causal factors other than (or in addition to) ageing are involved. Other contributory factors might be inadequate dietary intake of energy and/or protein (either due to geriatric anorexia, malabsorption, gastrointestinal disorders, or use of medications that cause anorexia). The onset of sarcopenia secondary to a systemic disease (particularly of an inflammatory, malignancy or endocrine nature or due to advanced organ failure) is usually (but not always) referred to as cachexia [2, 3]. Obese individuals with sarcopenia are a subgroup with a particularly high risk of adverse outcomes; the evidence suggests that the co-existence of these conditions creates a synergy in the risk of developing multiple comorbidities [10, 11].

## Regulatory insights from the field of osteoporosis

Sarcopenia and osteoporosis share many contributory (and causative) factors. Both are consequences of biological ageing and both are associated with higher risk of mobility limitation, fractures and disability in the activities of daily living [12]. Indeed “sarcopenia can be considered for muscle, what osteoporosis is to bone” [13]. Thus, it might be expected that there should be a number of parallels in the development of the definitions of each these conditions and how this has led, in the case of osteoporosis, to the development of regulatory and treatment guidelines. Bijlsma and colleagues [14] identified a number of milestones in the development of the recognition of osteoporosis as a disease, including: coining the term (in 1830), the development of non-invasive imaging [dual-energy X-ray absorptiometry (DXA)] (1987), the increasing pharmaceutical interest (linked to the development of putative anti-osteoporotic agents) and public awareness of the increased fracture risk with age (1980’s), the conceptual definition (1990), the operational definition (1994) and risk stratification (2008). It could be argued that for sarcopenia, we are at the stage of pharmaceutical interest, but before widespread public awareness; the crucial next step will be agreement on an operational definition with accepted thresholds of normal/abnormal muscle mass and function, and how these vary by gender and ethnicity.

Both bone and skeletal muscle are in a state of dynamic equilibrium; a constant process of breakdown and reconstruction. Muscle mass can be increased by resistance exercises, but if it is not optimally used it will be subsequently lost, since high muscle mass requires a relatively greater maintenance energy expenditure. A major difference with bone is that muscle mass can be lost and rebuilt fairly rapidly, at least in younger adults, but this ability to rebuild muscle diminishes significantly in older individuals [15]. Importantly, muscle constitutes an important reserve of protein that can be called upon in periods of undernutrition [16].

After a peak and a plateau phase in tissue growth, senescence steps in [17]; a process of biological ageing resulting in the gradual deterioration of bone density, muscle mass and strength. The loss in muscle mass is shaped by the decline in the production of growth and anabolic sex hormones, although the exact relationship between hormone status and muscle function is complex both in men and women [18, 19]. This contributes to the declining muscle strength [20] but is not completely explanatory [10, 21]. An important factor here is concomitant obesity [10, 22], since this will exacerbate the drop in anabolic hormone production [23] and negatively impact muscle quality [24], increasing the risk of sarcopenia in both men and women [11, 25]. The decline in muscle mass and strength in the majority of older individuals engenders no major hazard, but for some individuals the decline is severe, leading to a downward spiral of reduced mobility, frailty, increasing comorbidity risk and premature death.

A conceptual definition of osteoporosis was finally achieved at an international consensus conference held in Hong Kong in March 1993 [26]. An international consensus on a conceptual definition of sarcopenia still seems hesitant; the newer definitions, which introduce the notions of reduced mass, strength and function, are however gaining ground.

## Operational definitions

### Osteoporosis

The definition of osteoporosis was operationalized in 1994 [27], as being bone mineral density (BMD) T-score (measured using DXA) of −2.5 or lower (i.e. at least 2.5 standard deviations below average BMD of healthy young individuals). This threshold provides an indicator that defines individuals with a significantly greater fracture risk than the population average [28]. A diagnosis of osteoporosis therefore indicates an elevated risk of fracture and could lead to the prescription of an anti-osteoporotic agent. The risk of fracture, however, depends also on other clinical factors, such as prior fracture history, BMI, glucocorticoid use, family history, etc. risk also varies markedly in different countries. For these reasons, the decision whether or not to prescribe an anti-osteoporotic agent is frequently done using a risk algorithm (e.g. FRAX) with or without the incorporation of a BMD value [29]. Thus, the original biological marker, BMD, has lost some of its diagnostic relevance to the risk algorithms.

### Sarcopenia

Unlike the relatively straight-forward measurement of BMD and its comparison to a reference, muscle mass can be estimated by a variety of techniques and there are numerous methods for adjusting the result for body size and corpulence [7]. As a first step towards an operational definition of sarcopenia, the EWGSOP group argued the case for using three well-researched and measures of muscle mass or function: gait speed, hand grip strength (HGS) and appendicular lean mass (ALM); they also proposed a set of thresholds (cut-points) for each that could be indicative of a pathological condition.

More recently, the challenge was taken up by the research team for the Foundation for the National Institutes of Health (FNIH) Sarcopenia Project (FNIH-SP) which has now published a set of thresholds based on an extensive reanalysis of existing studies in sarcopenia [30]. Like the EWGSOP, the FNIH-SP group considered that gait speed, HGS and ALM are key measures of muscle mass and function which can be used for a diagnosis of sarcopenia. This group were of the opinion, however, that slow gait speed (i.e. mobility impairment) is more of a primary outcome of low muscle mass and strength and is not (necessarily) part of the diagnostic process. The research team used this “outcome” to determine threshold values for the two other criteria, first in cross-sectional analyses, and then using the values obtained in a predictive manner on the longitudinal data. The project pooled nine studies in older, community dwelling, individuals (*n* = 26,625 assessable participants) having available data on HGS, gait speed and body composition using DXA [30]. The average age of the cohort was 75.2 (standard deviation: ±6.1) years for men and 78.6 (±5.9) years for women, with a high prevalence of obesity in both genders.

## Diagnostic tests

### Gait speed

The clinically relevant threshold for gait speed chosen by the EWGSOP consensus and the FNIH-SP group was 0.8 m/s [2, 30]; below this level there is a strong association with reduced survival and increased risk of disability [31–34]. Both groups, and most other studies, have opted to use the same cut-off for both men and women. It is clear from various sources, however, that men walk faster than women even at advanced ages [35, 36], suggesting that having a single cut-off might overestimate the number of women with low lower-body physical capacity and underestimate the number of men. It is likely that different thresholds may also have to be made for different ethnic groups [37, 38].

### Handgrip strength

The FNIH-SP analysis [39] identified two threshold values each for men and for women; thus defining three strength groups: low, intermediate and high. For men these values were <26 and <32 kg (rounded to nearest whole value) and for women were <16 and <20 kg. The proportions of mobility disability observed in the three groups were, for men: 40, 21 and 6 %; and for women: 51, 36 and 20 %, respectively. The relevant low strength values of HGS suggested by EWGSOP were <30 kg for men and <20 kg for women.

It has previously been determined that body height is positively correlated with muscle strength (even after adjusting the dynamometer for hand size) and therefore should be an adjustment factor (fat mass should also be considered as an adjustment factor) [40]. The FNIH-SP group examined the effect of including this and other anthropomorphic factors in their classification and regression tree analyses to identify the most appropriate model for the prediction of slow gait speed, including the ratio of strength to body size (grip strength/height, grip strength/height^2^, grip strength/weight, and grip strength/BMI); only the adjustment by BMI appeared to have better predictive value, and only in women [41]. For the men, statistically significant interactions were observed for the adjustments on height (a stronger association between weakness and slow walking in taller men) and on age (a stronger association in the 65–79 year age group than in the 80+ years group). At present, the FNIH-SP group has advocated not adjusting HGS on anthropomorphic criteria.

### Appendicular lean mass

The FNIH-SP analysis [42] found that appendicular lean mass (ALM; the sum of the lean mass of both arms and both legs) was significantly and positively correlated with grip strength in men and in women. Using the first level thresholds of HGS as a definition of weakness (<26 and <16 kg in men and women, respectively), the study found an ALM threshold for men of <20 kg (rounded value), below which the prevalence of weakness was 18 % and above which was 2.5 %. For women, two ALM thresholds were identified, one at <12 kg and another at <15 kg, but for simplicity the lower of the two was ignored in further analysis. The prevalence of weakness in women with ALM <15 kg was 30 and 11 % if above this threshold.

Since, ALM varies according to body size, it is often indexed to the square of body height and thresholds applied by, for example, a rule of two standard deviations below a reference population [43] (giving cut-offs of <5.5 kg/m^2^ for women and <7.25 kg/m^2^ for men as advocated by EWGSOP). The FNIH-SP group tested various adjustments on anthropomorphic variables in their analyses, including ALM adjusted for height, or height squared, or weight, or BMI, or total body fat; as well as leg lean mass (LLM) adjusted for each of the above variables. Amongst these, the strongest associations between the other measures of muscle strength/performance were found when ALM was adjusted for (divided by) BMI (ALM_BMI_). For men, the ALM_BMI_ threshold was <0.789 (giving a group with a prevalence of weakness of 11.8 %); for women the threshold was <0.512 (giving a group with a prevalence of weakness of 31.0 %). In predictive analyses, these thresholds were associated with higher odds of mobility impairment for both men and women. ALM_BMI_ was not as strongly associated with incident mobility deficit as weakness, but it did significantly predict incident mobility impairment. This novel method of adjusting the measurement of lean mass probably explains in large part the differential rates of diagnosis of sarcopenia with respect to EWGSOP criteria [44] and will need further confirmation. Previous studies have shown that most of the inter-individual variation in ALM in persons of a similar age can be explained by height and weight (leaving aside gender and racial differences), and after controlling for these, ALM decreases with age by about 0.4 kg/decade in women and 0.8 kg/decade in men [45]. It is clear, however, that fat mass is very relevant to muscle quality [22, 46] and to the decline in gait speed [36]. So it is somewhat unexpected that the FNIH-SP researchers did not find any of the obesity measures selected as primary discriminators of weakness in the database and therefore abandoned obesity as being explanatory.

### Conclusions on proposed operational definitions of sarcopenia

The FNIH-SP group studies provide thought provoking correlations and warrant further investigation on the adjustment of measured values according to baseline variables such and body size and composition. It is interesting that here as well as in another recent study [47], the strongest correlations between measured parameters and “outcomes of sarcopenia” were not always the same in men and women. In the study by Scott and colleagues [47], the baseline data of an adult cohort (*n* = 1100) were analysed using various definitions of sarcopenia and the results correlated with the 5-year falls risk scores. The strongest correlations were men classified with sarcopenia according to anthropometric definitions (ALM corrected for height, weight or a residual), and women classified with sarcopenia according to performance-based definitions (HGS and lower-limb strength). Thus, there may be different processes in ageing between men and women that should be taken into account.

Comparing the rates of positive identifications obtained by the FNIH-SP threshold with previously suggested thresholds, suggests that the newer thresholds might be too severe. For example, applying the proposed HGS and ALM_BMI_, thresholds to the FNIH-SP pooled population resulted in just 1.3 % of the men and 2.3 % of the women being classified as “sarcopenic” and if the gait speed criterion was included then the yield was even lower [44]. Using the EWGSOP criteria on the same pooled dataset, the group sizes were 5.3 and 13.3 %, for men and women, respectively [44]. Testing the various thresholds suggested by EWGSOP on a Belgian cohort aged 65 years or more, Beaudart and colleagues found that the prevalence of sarcopenia (men and women) varied from 9 to 18 % [48]. When the analysis was carried out by age group, a higher incidence of sarcopenia was evidence with increasing age. Bischoff-Ferrari and colleagues [49] have also explored the consequences of using different definitions of sarcopenia to prospectively identify community-dwelling seniors who have a greater risk of falling, with the result that the strongest association was found using thresholds based on ALM corrected for height squared, although the EWGSOP definition was also strongly predictive. Clearly the choice of thresholds will significantly affect the size of the affected population, but the important data that are missing, concern the overall risk in the observed sample of individuals in terms of severe outcomes (major mobility impairment, falls, fracture, nursing home admissions, mortality) over the short- and long-term. Indeed, even if more and more data suggest that sarcopenia is associated with poor health outcomes, the methodology including the definition of sarcopenia differs widely between studies. More work is needed to characterise the hazard rates in a group of “sarcopenic” individuals.

## Target populations and study design for regulatory studies in the treatment of sarcopenia

The selection of patients for clinical research in the context of regulatory filing depends on the aims of the project and its stage in the development process (illustrated in supplementary data, Fig. 1). At early stages of development, the selection of the research population is more restrictive to reduce the possibility of confounding, but as the development process advances and different subgroups with specific comorbidities are integrated, then the population should broaden. Considerations for target populations for pharmaceutical trials in sarcopenia have been made previously [50, 51] and most recently by Vellas and colleagues [52]. It might also be added that prevention of sarcopenia in high-risk “pre-sarcopenic” individuals could be an achievable long-term goal. The EWGSOP suggested that a pre-sarcopenia stage could be characterised by the existence of low muscle mass alone. The concept of targeting such a population to slow or prevent the progression to sarcopenia might be considered as being similar to the recently completed LIFE study [53] in which older persons with a sedentary lifestyle and at high risk of mobility disability (Short Physical Performance Battery [SPPB] score <10) but able to walk 400 m in under 15 min, were randomised to an exercise programme compared with a health education programme. Although the inclusion criteria were quite different from that suggested by EWGSOP the endpoint of major mobility disability after a planned 31 months of follow-up appears pertinent.

The target population for the treatment of sarcopenia (or pre-sarcopenia) should be men and women aged 65 years or more [2, 30]. In the screening (diagnostic) process to recruit patients we advocate the use the EWGSOP criteria. The assessment of baseline parameters in the selected population of older individuals should as thorough as possible so that individual risk status can be assessed, as well as providing information on other more exploratory variables (examples provided in supplementary data Table 1). For any drug development plan, it will be important to engage in dialogue with regulators during its design to validate recruitment decisions and exclusion criteria.

### Phase II

In a phase II programme (about 300 individuals), exploratory and proof of concept studies are followed by dose-ranging and short-term efficacy studies. The study population must be relatively homogeneous with the exclusion of a range of comorbid conditions to reduce confounding of the diagnosis or efficacy assessment (e.g. major endocrine, pulmonary, cardiac neurological or renal conditions, as well as chronic inflammatory rheumatic conditions).

A recently published international phase II study in sarcopenia, which was designed to test the effects of the selective androgen receptor modulator [54], selected women of 65 years old or more, with low ALM (ALM/height^2^ versus reference), self-reported mobility disability and an SPPB score between 4 and 9. The exclusion criteria included an extensive list of comorbidities (Supplementary data, exclusion criteria). The research team reported a randomisation rate of 29 % of those screened (170 out of 592).

### Phase III

The phase III study population should be a logical continuation of the phase II programme so as to provide convincing data on the benefit–risk balance of the study drug or intervention in the intended target population. There should be no upper age limit for the included population [55]. Special subpopulations should be considered, for example persons with previous hip fracture, or concomitant condition or those in particular settings (e.g. nursing home or acute care).

## Study design

The placebo-controlled, parallel arm, double-blind trial is the mainstay of regulatory study design and is suitable here. A stable baseline should be ensured with a relatively long run-in phase before treatment start (e.g. 4–6 weeks) during which activity diaries could be monitored and any dietary failings or anaemia corrected [56, 57]. Studies should, as much as possible, have similar time points for assessment (e.g. 1 month, 3 months, 6 months, 1 year) so that comparisons between studies, and thus data pooling, or meta-analyses, are facilitated. For studies in pre-sarcopenic patients that aim to slow or prevention the progression to sarcopenia, longer follow-up durations will probably be necessary.

## Outcome measures

The primary endpoints in the exploratory and dose-ranging stages of a phase II programme are likely to be biological and/or pharmacodynamic parameters. The European Drug Agency (EMA) has emphasized that it is important to perform modelling of population pharmacokinetics as well as specific pharmacokinetic studies in the very elderly. As the development proceeds to short-term efficacy studies, then the choice of efficacy measures needs to be taken. The relevant functional outcome measures in this phase are gait speed, lower leg strength and possibly other more functional tests such as SPPB [58] and Timed Up and Go (TUG) [59], which have their minimal clinically meaning differences already established.

A phase III pivotal study needs to show substantial evidence that a drug will have the desired effect in the proposed labelling; the primary endpoint should be a direct measure of either: improved survival; a benefit detectable by the patient (e.g. improvement in functional capacity); or a reduction in the risk of developing a condition (e.g. mobility disability), or disease complication that is itself apparent to the patient and undesirable. How any improvement in physical functioning might lead to reduced costs for healthcare systems will be important to support the submission [60].

It is challenging to identify a single clinical endpoint that is sufficiently robust and therefore more that one measure should be selected (although the question of a co-primary endpoint is debatable). Possible outcome measures are listed in Table 1. Longer duration exercise tests have a stronger case for being a meaningful function outcome and are highly relevant to older patients for whom crossing the road may be a risky enterprise [35]. There are two well-known walking protocols of similar discriminatory efficacy: the 6-min walk test and the 400 m walk test. The result of the 6-min walk test is the total distance walked over 6-min has proved popular in studies of cardiac rehabilitation. The result of the 400 m walk test [61] measures the time taken to walk that distance and has a high test–retest reliability [62]. It can also be used as a binary outcome (yes/no result) whether the person can complete the test within 15 min or not. Stair climbing can be discriminatory and particularly when “loaded”, i.e. carrying a bag in each hand with a combined weight of 20–25 % of body weight [63]. Tests of thigh muscle strength (knee extension) have also shown their value in research [64, 65] but specialised equipment is required. Muscle fatigue is an important aspect of muscle performance and various methods exist to measure it [66–68], but no clear choice stands out at this stage. Any measure of muscle performance must clearly take into account the capacity of the sample population to perform it, since some can be quite challenging. Improvement under treatment using shorter tests such measures as gait speed, SPPB and TUG test will probably only be considered as supporting evidence, since these surrogates have no proven direct relationship to clinical benefit.

**Table 1:** Some outcome measures proposed for phase III regulatory studies

Outcome measure	Test
Falls	Incident falls or perhaps incident recurrent falls (i.e. ≥2 falls); but unless the person is fitted with an accelerometer this is a patient-reported outcome
Major mobility disability	Incapacity to complete 400 m walk test within 15 min (i.e. <0.45 m/s)
ADL disability	Activities of daily living (ADL)
Patient-reported outcomes	The 36-item short-form (SF-36) of the Medical Outcomes Study (a generic QOL tool) EQ-5D (a generic QOL tool) A specific age-related tool (see reference [8])

The assessment of falls has been suggested as a possible outcome in sarcopenia studies. Up until now this type of measure has relied heavily on the patient recording in a diary the occurrence of falls and therefore of uncertain reliability. Recently, however, with the development of mobile electronic devices [69], smart wearable sensors and motion detectors are becoming much cheaper and more reliable for research purposes [70], potentially allowing a more objective assessment.

For complete assessment of the benefits of any intervention it is important to provide evidence of the impact in terms of health status and quality of life and it will be essential to place any clinical trial data in the context of a comprehensive global assessment in older people with chronic illness [71]. There are a number of well-known patient-reported outcome (PRO) instruments available, however, as reported previously [8] the validity of many of these PRO instruments is poorly documented in older populations. Probably the most suitable instrument at the present time is the SF-36 [72], since it adequately covers the three key domains (physical/occupational function, social health/integration, and mental health/psychological state), is not too onerous to complete, is well known and has proved suitable in more than one sarcopenic/frail cohort [73, 74]. The EQ-5D [75] has also been used as a PRO instrument in sarcopenia and under-nutrition research. These remain, however, “generic” measures and as such may be relatively insensitive to some perceived changes [8].

## A core outcome set

**Fig. 1:**
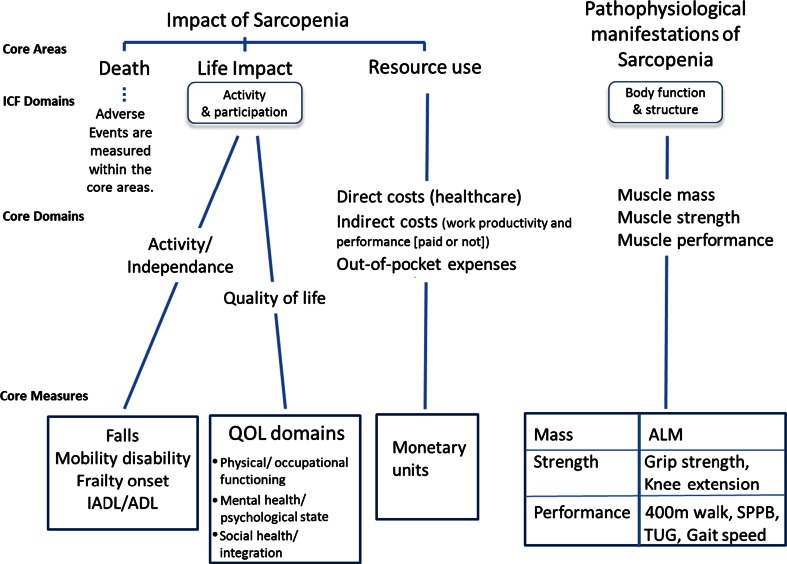
The application of the OMERACT filter 2.0 for the definition of core outcomes for sarcopenia. *ICF* International Classification of Functioning, Disability and Health framework of the World Health Organisation

The selection of a core outcome set to be used in all clinical trials of sarcopenia would be an important goal of a clinical guideline. Even if the main endpoints might vary in different studies, having a core outcome set would enhance trial comparison and therefore improve the evidence base, as has been shown for rheumatological diseases [76, 77]. Recently, the Core Outcomes Measure in Effectiveness Trials (COMET) initiative [78] was established to provide a database of such outcome sets and help their development and utilisation. Outcome measures in rheumatology (OMERACT), has developed a tool, “filter 2.0”, which outlines the intellectual process of deciding first what to measure (domains), and then how to measure it (instruments) [79]. This tool suggests that three core areas that should always be addressed in a core set: death, life impact and pathophysiological manifestations of the disease; a fourth area, resource use, is strongly recommended. The resulting sets of domains and measurement instruments should then pass through a consensus selection process. Figure 1 illustrates this process with reference to sarcopenia.

The impact area ‘death’ is invariant in the filter and an important outcome measure. Adverse events are also important and researchers should decide if specific adverse events need to be monitored as part of the core set. ‘Life impact’ includes assessments of mobility/disability which, for sarcopenia, would include the strong endpoints of falls and fractures, reduced mobility, frailty onset (e.g. Fried criteria [80], Instrumental Activities of Daily Living, (Basic Activities of Daily Living [81]), as well as PRO instruments relating to quality of life. ‘Resource use’ is a core area of interest in later stage development. The domains are fairly self-explanatory and the relevant metric is monetary units [although unitary use of health care resources (specialist care, home care, admission to nursing home) could be considered]. It should be added that an important (and growing) proportion of older people are employed in some sense or another, notably in voluntary work (e.g. in libraries, caring for partners or family members). ‘Pathophysiological manifestations of sarcopenia’ includes direct measures of muscle function and structure (see Fig. 1).

A strong recommendation of ESCEO is therefore that a core set be developed for use in sarcopenia, i.e. the selection of at least one applicable instrument for the assessment of each core domain.

## Discussion

Healthy ageing is a major goal of both European and international initiatives [82, 83]; with the increasing lifespan in western populations, it is becoming more and more important to prevent disability for as long as possible. Important advances have been made in defining sarcopenia, particularly with the consensus definitions of 2010 and the recent input from the FNIH Sarcopenia Project and there is now a “broad support for the existence of a clinically important condition of low muscle mass and weakness” [30]. The evidence suggests that sarcopenia is a treatable condition. While one solution might be to prescribe an exercise programme, many older individuals lack the necessary physical and mental energy to pursue it or may be physically impaired and so unable to participate. For these individuals a pharmaceutical product could be of help and indeed numerous agents currently under assessment for the treatment of sarcopenia, including testosterone, dehydroepiandrosterone, oestrogen, growth hormone, ghrelin, angiotensin converting enzyme inhibitor, eicosapentaenoic acid and ryanodine receptor modulators [6]. Other therapies include various oral nutritional supplements (proteins, amino acids, vitamin D, etc.).

The public health problem associated with sarcopenia is likely to be substantial [84] but, because of the uncertainty in the diagnosis of sarcopenia its prevalence and epidemiology, the size of the problem is far from clear. In a much quoted paper from 2004, the costs attributable to sarcopenia (with a diagnosis based on muscle mass index only) using risk estimates of progression to disability and estimations of the cost burden by disability scoring, were estimated at $18.5 billion per year in the USA (range $11.8–26.2 billion) [85]. For comparison the attributable costs of osteoporosis according to the American National Osteoporosis Foundation, is $13.8 billion a year, affecting approximately 2 million Americans (80 % of them women).

As well as preventative physical activity programmes, oral supplements and perhaps pharmaceutical interventions, older adults need to be better educated in the importance of healthy nutrition and body weight. Adults need to know the importance of “sufficient” muscle mass and muscle function and the dangers associated with increase fat mass; to understand that if the ratio of fat and muscle increases in favour of fat, they risk not being able to get up from a chair or walk safely in old age, and having a high chance of developing other problematic chronic conditions. Effective strategies for pre-sarcopenia and sarcopenia will need to combine, nutritional support and education to reduce sedentary behaviour and encourage exercise. The benefits of pharmaceutical intervention in resistant cases will then have to be weighed against the risks in a population likely to be poly-medicated. The development and evaluation of complex interventions (or “multifactorial interdisciplinary” interventions) will be challenging [86].

### A research agenda

There are still numerous gaps in our knowledge, particularly concerning risk assessment. It would be instructive to build risk models similar to those for osteoporosis, starting with the person’s age, sarcopenia-related risk factors and other risk factors and assess the outcome for this individual over time. Although it is argued that age may have little relevance in a diagnosis of frailty [87], it remains a reasonable approximation to biological age and its associated hormonal changes. Age is also a good predictor of osteoporotic fracture risk [88].

The value of indexing threshold values for sarcopenia measures and outcomes needs to be further investigated, as does the need for sex-dependent values for gait speed. Such an evaluation would best be achieved using a risk-based analysis for one of the discussed strong clinical endpoints.

A consensus core outcome set would bring standardization and comparability to research in sarcopenia and therefore would help improve the evidence base for health care [77]. A selection of the proposed outcomes and the measurement techniques is required and some issues could be resolved by launching a consultation with a Delphi type voting process.

It is very important that thresholds should be selected using the best evidence so they can be widely accepted. In the light of new evidence, they can be modified; as pointed out by Studenski and colleagues [30], the currently accepted threshold values for blood pressure, blood sugar concentration, or cholesterol levels, used respectively, to diagnose hypertension, diabetes and hyperlipidemia, were all selected empirically from a continuous graded relationship of risk of serious adverse events and all have evolved over time.

## Electronic supplementary material

Supplementary material 1 (DOCX 15 kb)

Supplementary material 2 (DOCX 73 kb)

Supplementary material 3 (DOCX 17 kb)
