# Supplementary material for: Recommendations for the conduct of clinical trials for drugs to treat or prevent sarcopenia
Source: Aging Clin Exp Res. 2015 Dec 30;28:47–58. doi: 10.1007/s40520-015-0517-y (PMC4768478; doi:10.1007/s40520-015-0517-y)
Supplement: Supplementary file 1 — Supplementary material 1 (DOCX 15 kb) [file 40520_2015_517_MOESM1_ESM.docx]

**Supplementary data –Exclusion criteria examples**

**Exclusion criteria in the phase II study in sarcopenia by Papanicolaou et al (2012)[54]**

Participants with any of the following conditions/medicinal product use:

1. Uncontrolled thyroid disease
2. Uncontrolled diabetes
3. Cancer
4. Uncontrolled hypertension
5. Chronic lung disease
6. End-stage organ disease
7. Neuromuscular or neurologic disease causing muscle weakness (Parkinson’s disease, amyotrophic lateral sclerosis, stroke affecting lower extremity function, muscular dystrophy, epilepsy, multiple sclerosis)
8. Signs or symptoms of cardiovascular disease
9. Unstable angina (NYHA)
10. Class III or IV congestive heart failure
11. Rheumatoid arthritis
12. Conditions that cause significant muscular or joint pain or significantly limit mobility, including polymyalgia rheumatica, polymyositis, and fibromyalgia
13. Conditions or concomitant medications that may impact the assessment of improved muscle strength and function
14. Use of myoanabolic agents and strong inhibitors or inducers of CYP3A4
