# Supplementary material for: Recommendations for the conduct of clinical trials for drugs to treat or prevent sarcopenia
Source: Aging Clin Exp Res. 2015 Dec 30;28:47–58. doi: 10.1007/s40520-015-0517-y (PMC4768478; doi:10.1007/s40520-015-0517-y)

**Supplementary data - Figure 1**

**Figure 1: Patient populations for regulatory studies according to the stage of project development and to the licensed indication and contraindications**


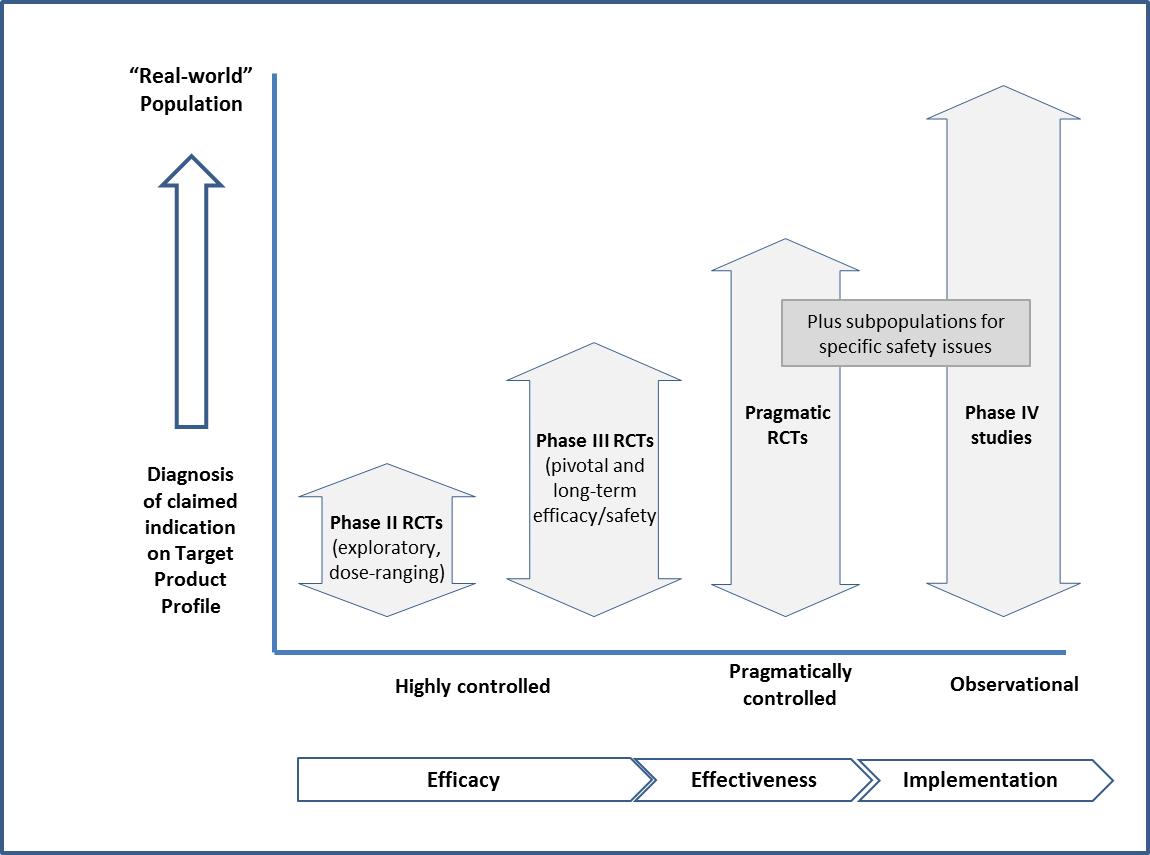

Supplement: Supplementary file 2 — Supplementary material 2 (DOCX 73 kb) [file 40520_2015_517_MOESM2_ESM.docx]
