# Supplementary material for: Recommendations for the conduct of clinical trials for drugs to treat or prevent sarcopenia
Source: Aging Clin Exp Res. 2015 Dec 30;28:47–58. doi: 10.1007/s40520-015-0517-y (PMC4768478; doi:10.1007/s40520-015-0517-y)
Supplement: Supplementary file 3 — Supplementary material 3 (DOCX 17 kb) [file 40520_2015_517_MOESM3_ESM.docx]

**Supplementary data - Table 1**

**Table 1: Examples of possible variables and tests to characterise the older populations included in clinical trials** (in addition to gender, ethnicity, demographic and anthropomorphic parameters)

| **Explanatory variable group** | **Possible tests** |
| --- | --- |
| **Lifestyle** | Smoking status, daily physical activity level, professional status (retired, full- or part-time work) [1] |
| **Cognitive status** | Montreal Cognitive Assessment [2] or  Folstein’s Mini-Mental State Examination |
| **Comorbidity** | Cumulative Illness Rating Scale for geriatrics CIRS-G [3], knee pain |
| **Nutritional status** | Mini nutritional assessment [4], SNAQ65+ [5] |
| **Hormone status** | Testosterone [6,7], Insulin-like growth factor 1 [8], vitamin D [9] |
| **Genetics** | Angiotensin-converting enzyme (ACE) genotype [10], telomere length/dysfunction [11], (Birth weight [12]) |
| **Muscle physiology** | A wide variety of biomarkers have been suggested including agrin expression (implicated in the stability of neuromuscular junction), myostatin expression (implicated in the mTOR signalling pathway of muscle development), satellite cell content or protein degradation [8,13,14] |

[1] Stenholm S, Westerlund H, Head J, et al. Comorbidity and Functional Trajectories From Midlife to Old Age: The Health and Retirement Study. *J Gerontol A Biol Sci Med Sci.* 2014;glu113.

[2] Nasreddine ZS, Phillips NA, Bedirian V, et al. The Montreal Cognitive Assessment, MoCA: a brief screening tool for mild cognitive impairment. *J Am Geriatr Soc.* 2005;53(4):695-9.

[3] Miller MD, Paradis CF, Houck PR, et al. Rating chronic medical illness burden in geropsychiatric practice and research: application of the Cumulative Illness Rating Scale. *Psychiatry Res.* 1992;41(3):237-48.

[4] Cuervo M, Garcia A, Ansorena D, et al. Nutritional assessment interpretation on 22,007 Spanish community-dwelling elders through the Mini Nutritional Assessment test. *Public Health Nutr.* 2009;12(1):82-90.

[5] Wijnhoven HA, Schilp J, van Bokhorst-de van der Schueren MA, et al. Development and validation of criteria for determining undernutrition in community-dwelling older men and women: The Short Nutritional Assessment Questionnaire 65+. *Clin Nutr.* 2012;31(3):351-8.

[6] Carcaillon L, Blanco C, Alonso-Bouzon C, et al. Sex differences in the association between serum levels of testosterone and frailty in an elderly population: the Toledo Study for Healthy Aging. *PLoS One.* 2012;7(3):e32401.

[7] Carcaillon L, Garcia-Garcia FJ, Tresguerres JA, et al. Higher levels of endogenous estradiol are associated with frailty in postmenopausal women from the toledo study for healthy aging. *J Clin Endocrinol Metab.* 2012;97(8):2898-906.

[8] Gonzalez-Freire M, de Cabo R, Studenski SA, Ferrucci L. The Neuromuscular Junction: Aging at the Crossroad between Nerves and Muscle. *Front Aging Neurosci.* 2014;6:208.

[9] Bischoff-Ferrari HA. Relevance of vitamin D in muscle health. *Rev Endocr Metab Disord.* 2012;13(1):71-7.

[10] Buford TW, Hsu FC, Brinkley TE, et al. Genetic influence on exercise-induced changes in physical function among mobility-limited older adults. *Physiol Genomics.* 2014;46(5):149-58.

[11] Hubbard JM, Cohen HJ, Muss HB. Incorporating Biomarkers Into Cancer and Aging Research. *J Clin Oncol.* 2014;32(24):2611-6.

[12] Sayer AA, Syddall HE, Gilbody HJ, et al. Does sarcopenia originate in early life? Findings from the Hertfordshire cohort study. *J Gerontol A Biol Sci Med Sci.* 2004;59(9):M930-M934.

[13] Sakuma K, Aoi W, Yamaguchi A. The intriguing regulators of muscle mass in sarcopenia and muscular dystrophy. *Front Aging Neurosci.* 2014;6:230.

[14] Schiaffino S, Dyar KA, Ciciliot S, et al. Mechanisms regulating skeletal muscle growth and atrophy. *FEBS J.* 2013;280(17):4294-314.
